# ﻿A new species of the *Amynthas corticis* group with support from mitogenomic data and a new record of *Metaphire agrestis* (Goto & Hatai, 1899) (Oligochaeta, Megascolecidae) in northeastern China

**DOI:** 10.3897/zookeys.1266.161903

**Published:** 2026-01-12

**Authors:** Anne Charis N. Han, Nannan Zhao, Nonillon M. Aspe, Christian Jay R. Nob, Yufeng Zhang, Donghui Wu, Huifeng Zhao

**Affiliations:** 1 Hebei Key Laboratory of Animal Diversity, College of Life Science, Langfang Normal University, Langfang 065000, China Northeast Normal University Changchun China; 2 State Environmental Protection Key Laboratory of Wetland Ecology and Vegetation Restoration, School of Environment, Northeast Normal University, Changchun 130117, China Langfang Normal University Langfang China; 3 College of Environment and Life Sciences, Mindanao State University at Naawan, Misamis Oriental, Naawan 9023, Philippines Mindanao State University at Naawan Naawan Philippines; 4 Key Laboratory of Wetland Ecology and Environment, State Key Laboratory of Black Soils Conservation and Utilization, Northeast Institute of Geography and Agroecology, Chinese Academy of Sciences, Changchun 130102, China Northeast Institute of Geography and Agroecology, Chinese Academy of Sciences Changchun China

**Keywords:** Earthworm, East Asia, pheretimoids, taxonomy

## Abstract

As part of a series of investigations on earthworm diversity conducted in northeastern China in recent years, a new species, *Amynthas dandongensis* Han & Zhao, **sp. nov.**, has been discovered. It belongs to the *A. corticis* species group characterized by having four pairs of spermathecal pores in segments 5/6/7/8/9. The new species mainly differs from the other members of the *A. corticis* species group by its relatively small body size and the absence of male pores and genital markings in both the spermathecal and male pore regions. The interspecific pairwise COI genetic distances between *A. dandongensis* Han & Zhao, **sp. nov.** and the other closely related species ranged from 15% to 22%. Phylogenetic analyses using mitogenomic coding genes further explored the relationships within the *A. corticis* species group, clarifying the position of *A. dandongensis* Han & Zhao, **sp. nov.** in the group. Additionally, a new record of a cosmopolitan earthworm, *Metaphire agrestis* (= *Amynthas agrestis*) (Goto & Hatai, 1899) has been investigated in China. New sequences from specimens collected in northeastern China were analyzed together with “*M. agrestis*” and “*A. agrestis*” sequences available from GenBank to further explore phylogeographic relationship. The dataset of all *M. agrestis* sequences showed intraspecific genetic distances of 0–1%, consistent with the observed morphological similarities. Haplotype network analyses also revealed no genetic isolation among different populations of *M. agrestis* (= *A. agrestis*). Moreover, re-examination of previous descriptions and illustrations of the male pores of *agrestis* indicates that this species should be assigned to the genus *Metaphire* Sims & Easton, 1872, rather than *Amynthas* Kinberg, 1867.

## ﻿Introduction

Earthworms, as prominent macro-invertebrate detritivores, significantly contribute to organic matter decomposition and nutrient cycling in soil ecosystems (Edwards et al. 2022). China has the richest earthworm diversity in East Asia, with family Megascolecidae Rosa, 1891 constituting more than 90% of the total earthworm species (Aspe 2016; Zhao et al. 2025).

Statistical data indicate a decline in megascolecid species diversity from southern to northern China, which is likely influenced by regional climatic variations in temperature and humidity. The higher diversity in southern regions may reflect more favorable environmental conditions for earthworms. However, the observed differences could also be partly due to insufficient sampling efforts in northern China for taxonomic studies (Jiang 2016).

Pheretimoids are the largest earthworm group in the Megascolecidae, characterized by having a perichaetine setal arrangement, a meronephridial excretory system, a single gizzard in VIII, a pair of racemose prostates opening through male pores in XVIII, and testes contained within testis sacs (Sims and Easton 1972). *Amynthas* Kinberg, 1867 and *Metaphire* Sims & Easton, 1972 are the most dominant genera in East Asia (Aspe 2016; Sato et al. 2023), especially in China (Jiang and Qiu 2018; Zhao et al. 2025). The distinction between these two genera is determined by the presence or absence of copulatory pouches (Blakemore 2009, 2010a; Nguyen et al. 2020). However, the definition of a “true” copulatory pouch remains a subject of debate (James 2005; James et al. 2005; Chang et al. 2008; Chang et al. 2014; Shen et al. 2019). A number of descriptions have been proposed for the organ, such as whether only the invagination of body walls is considered as copulatory pouches (e.g., James 2005; James et al. 2005), or whether intramural chambers and even the shallow indentations should be considered (Tsai et al. 2009; Shen et al. 2019). This confusion has led to the emergence of synonymies and recombination of species within these two genera (Jeratthitikul et al. 2017). Additionally, the ambiguity of some species’ taxonomic assignment between both genera has been a subject of considerable discussion. For instance, the species *tschiliensis* currently assigned to the genus *Metaphire* as *M. tschiliensis* (Michaelsen, 1928) has been previously referred to as *A. tschiliensis* in Tsai et al. (2000). However, Shen (2018) further confirmed and stated that “*tschiliensis* possesses copulatory pouches (Chen et al. 1975), which would place it within the *Metaphire*”. Other examples include the species *M. formosae* (Michaelsen, 1922) and *M. yuhsi* (Tsai, 1964). Originally described as *Pheretima formosae* and *P. yuhsi*, respectively (Michaelsen 1922; Tsai 1964), both species were later reclassified under the genus *Amynthas* by Sims and Easton (1972). Not until the re-inspection of the specimens by Chang and Chen (2005), that the two species have been re-assigned to *Metaphire* due to the presence of copulatory pouches in the male pores (Chang et al. 2008).

Meanwhile, the genus assignment of *Perichaeta agrestis* Goto & Hatai, 1899 is ambiguous. Some researchers have classified it within *Amynthas* (Sims and Easton 1972; Reynolds 1978; Easton 1981; Reynolds and Wetzel 2004; Hong and Kim 2005; Blakemore 2007a, 2010a, 2013a; Chang et al. 2016; Shekhovtsov et al. 2018; Schall et al. 2023), while others have placed it within *Metaphire* (Blakemore 2003; Sato et al. 2023). This discrepancy highlights the need for further taxonomic clarification.

Recent extensive sampling efforts in northeastern China have focused on pheretimoids (Han et al. 2024; Zhao et al. 2025). Notably, *M. liaoningensis* Han & Zhao, 2025 has been described in the region, and the species was identified based on morphological characteristics and mitogenomic data. This species represents a significant addition to the regional earthworm fauna of northeastern China (Zhao et al. 2025).

Resolving phylogenetic relationships based solely on mitochondrial COI is insufficient, particularly for species-complex such as *Eisenia nordenskioldi* (Eisen, 1978) (Qin et al. 2025) and *Drawida ghilarovi* Gates, 1969 (Liu et al. 2025). To enhance phylogenetic resolution, Chang and James (2011) suggested incorporating longer sequences from both mitochondrial and nuclear genes. Mitogenomic data have become increasingly effective for constructing high-resolution phylogenetic trees (Zhao et al. 2022; Sato et al. 2023). These approaches are especially relevant for pheretimoid species, where key taxonomic characteristics such as male internal structures and external pores of spermathecal organs are often lost through parthenogenesis (Blakemore 2010), complicating species delineation.

This study documents a subset from a broader dataset of pheretimoid earthworm species previously investigated in northeastern China. Here, we present a new species, *A. dandongensis* sp. nov., which belongs to the *A. corticis* species group. Our findings are supported by both morphological and molecular data. In order to ascertain its phylogenetic relationships with other pheretimoid species, mitogenomic data of *A. dandongensis* sp. nov. were incorporated. Additionally, we report the first record of the species *M. agrestis* (Goto & Hatai, 1899) in China and its current distribution. We also discuss the reassignment of this species to the genus *Metaphire*, based on a re-examination and comparison of male pore illustrations from previous specimens.

## ﻿Materials and methods

Earthworm specimens were collected from Jinjiangshan Park, Dandong Prefecture, Liaoning Province (40.1312°N, 124.3746°E, 40 m elevation) in July 2023. Specimens were obtained through digging and hand sorting, with particular attention to areas near surface castings. They were preserved in 100% ethanol in the field and stored at –20 °C in the laboratory.

Morphological examination of external and internal structures was conducted using a ZEISS stereomicroscope equipped with ZEN 3.3 Pro software for image capture. This facilitated the identification and measurement of small organs and other morphological features. Taxonomic assignments and generic diagnoses were based on the criteria outlined by Sims and Easton (1972).

For molecular analysis, total genomic DNA was extracted from the muscle tissue of the posterior part of nine earthworms using the TIANGEN Genomic DNA Kit (China) respectively, following the manufacturer’s instructions. The cytochrome c oxidase subunit I (COI) gene was amplified using polymerase chain reaction (PCR). The reaction mixture (total volume 25 μL) contained 1 μL DNA template, 17.25 μL sterile distilled water, 2.0 μL dNTPs, 2.5 μL buffer, 0.25 μL Easy Taq Polymerase (TransGen Biotech Co., Ltd., Beijing, China), 1.0 μL forward primer LCO1490 (5’-GGTCAACAAATCATAAAGATATTGG-3’) (Folmer et al. 1994), and 1.0 μL reverse primer COIE (5’-TATACTTCTGGGTGTCCGAAGAATCA-3’) (Bely and Wray 2004). The PCR cycling conditions were as follows: initial denaturation at 95 °C for 5 min, followed by 35 cycles of denaturation at 95 °C for 30 s, annealing at 51 °C for 30 s, and extension at 72 °C for 30 s, with a final extension at 72 °C for 5 min. Positive PCR products were sequenced by Tianyi Huiyuan Biotechnology Co., Ltd. (Beijing, China) using the Sanger method. The COI sequences were submitted to GenBank (accession numbers are given in Table 1).

**Table 1.** Specimen information of *Amynthas dandongensis* sp. nov. with species belonging to the *A. corticis* group indicated with an asterisk (*) and *M. agrestis* analyzed for genetic distance comparison, and other pheretimoid species for phylogenetic analysis using mitogenomes in this study.

Genetic marker	Specimen ID	Species	Accession number	Country	Source
mitogenome	–	A. carnosus*	KT429008	China (mainland)	Zhang et al. 2016
mitogenome	–	A. corticis*	KM199290	China (mainland)	Zhang et al. 2016
mitogenome	533R60_01	*A. dandongensis* sp. nov.*	PV588238	China (mainland)	this study
mitogenome	–	A. divergens*	LC726507	Japan	Sato et al. 2023
mitogenome	–	A. divergens*	LC726542	Japan	Sato et al. 2023
mitogenome	–	*M. guillelmi*	PQ164794	China (mainland)	Zhao et al. 2025
mitogenome	–	*M. guillelmi*	KT429017	China (mainland)	Zhang et al. 2016
mitogenome	–	*M. liaoningensis*	PP507115	China (mainland)	Zhao et al. 2025
mitogenome	–	*M. liaoningensis*	PP503847	China (mainland)	Zhao et al. 2025
mitogenome	–	*M. tschiliensis*	PP503849	China (mainland)	Zhao et al. 2025
mitogenome	–	*M. vulgaris*	KJ137279	China (mainland)	Zhang et al. 2016
mitogenome	–	*Polypheretima andresi*	PP266606	Philippines	Aspe et al. 2025
COI	–	A. amis*	JX290430	China (Taiwan)	Shen 2012
COI	–	*A. amis*	KP030700	China (mainland)	unpublished
COI	–	A. corticis*	w33	South Korea (Jeju)	Blakemore
COI	–	A. corticis*	JX290431	China (Taiwan)	Shen 2012
COI	–	A. corticis*	LC703220	Japan	Sota et al. 2023
COI	533R57_01	*A. dandongensis* sp. nov.*	PV453020	China (mainland)	this study
COI	533R58_01	*A. dandongensis* sp. nov.*	PV453021	China (mainland)	this study
COI	533R60_01	*A. dandongensis* sp. nov.*	PV453022	China (mainland)	this study
COI	533R63_01	*A. dandongensis* sp. nov.*	PV453023	China (mainland)	this study
COI	–	A. diffringens*	MT444902	India	Vabeiryureilai et al. 2020
COI	–	A. divergens*	LC722899	Japan	Sota et al. 2023
COI	–	A. exiguus exiguus*	ON314447	Vietnam	Lam et al. 2021
COI	–	A. exiguus*	KU565189	Thailand	Jeratthitikul et al. 2017
COI	–	A. heterochaetus*	EF077559	China (mainland)	Huang et al. 2007
COI	–	A. lini*	DQ224174	China (Taiwan)	Chang et al. 2007
COI	–	A. maximus*	MG450707	China (mainland)	Dong et al. 2019
COI	–	A. penpuensis*	KC897070	China (Taiwan)	Shen et al. 2014
COI	–	A. shengtangmontis*	MG450709	China (mainland)	Dong et al. 2019
COI	–	A. shengtangmontis minusculus*	MG450710	China (mainland)	Dong et al. 2019
COI	–	A. stricosus*	KF205972	China (mainland)	Sun 2013
COI	–	A. szechuanensis*	KF205477	China (mainland)	Sun 2013
COI	–	A. taiwumontis*	KC897068	China (Taiwan)	Shen et al. 2014
COI	–	A. tortuosus*	MG450708	China (mainland)	Dong et al. 2019
COI	–	A. uvaglandularis*	MK251494	China (Taiwan)	Shen et al. 2019
COI	–	A. wujhouensis*	JQ936607	China (Taiwan)	Shen et al. 2013
COI	–	A. wulinensis*	DQ224182	China (Taiwan)	Chang et al. 2007
COI	–	A. yunlongensis*	KF179581	China (mainland)	Sun 2013
COI	–	A. carnosus carnosus*	PP067671	China (mainland)	Han et al. 2024
COI	–	A. carnosus roki*	w56	South Korea	Blakemore and Lee, 2013
COI	–	A. aquilonius*	MK251481	China (Taiwan)	Tsai et al. 2001
COI	–	A. dinghuensis*	KU232793	China (Taiwan)	Shen et al. 2016
COI	–	A. dongjuensis*	KC897053	China (Taiwan)	Shen et al. 2014
COI	–	A. dispersus*	KY774375	China (mainland)	Sun et al. 2018
COI	–	A. gageodo*	G1	South Korea	Blakemore et al. 2012
COI	–	A. huapingensis*	OL693179	China (mainland)	Li et al. 2024
COI	–	A. taoyuanensis*	PP497095	China (mainland)	Jin et al. 2024
COI	–	A. xiangtanensis*	PP497097	China (mainland)	Jin et al. 2024
COI	–	*M. formosae*	PQ787826	China (Taiwan)	unpublished
COI	–	*M. formosae*	AY960807	China (Taiwan)	Chang et al. 2008
COI	533R71_02	*M. agrestis*	PV453015	China	this study
COI	533R72_03	*M. agrestis*	PV453016	China	this study
COI	533R74_01	*M. agrestis*	PV453017	China	this study
COI	533R74_02	*M. agrestis*	PV453018	China	this study
COI	533R75_02	*M. agrestis*	PV453019	China	this study
COI	–	*M. agrestis*	LC726525	Japan	Sato et al. 2023
COI	–	*M. agrestis*	LC721145	Japan	Sato et al. 2023
COI	–	*M. agrestis*	LC722900	Japan	Sato et al. 2023
COI	–	*M. agrestis*	KY750702	Russia	Shekhovtsov et al. 2018
COI	–	*M. agrestis*	KX400710	Russia	Shekhovtsov et al. 2018
COI	–	*M. agrestis*	KX400698	Russia	Shekhovtsov et al. 2018
COI	–	*M. agrestis*	AB542605	Japan	unpublished
COI	–	*M. agrestis*	AB542606	Japan	unpublished
COI	–	*M. agrestis*	AB542607	Japan	unpublished
COI	–	*M. agrestis*	AB542608	Japan	unpublished
COI	–	*M. agrestis*	AB542609	Japan	unpublished
COI	–	*A. agrestis*	PP118218	USA	Owen and Wilson 2024
COI	w48	*A. agrestis*	w48	South Korea	Blakemore 2013b
COI	w49	*A. agrestis*	w49	South Korea	Blakemore 2013b
COI	–	*A. tokioensis*	OR355673	Canada	Bennett et al. 2024
COI	–	*M. communissima*	AB542628	Japan	unpublished
COI	–	*M. communissima*	AB542627	Japan	unpublished

Genetic distances were calculated using the Kimura 2-parameter (K2P) model (Kimura 1980) performed in MEGA5 (Tamura et al. 2011) based on COI sequences from this study and other relative pheretimoid species that were downloaded from the GenBank (Table 1). For representative members of *A. corticis* species group, a neighbor-joining analysis was conducted using the K2P model in MEGA5 with 1,000 bootstraps to evaluate the robustness of clades. The molecular indices, including the sequence polymorphic sites (S), number of haplotypes (Hn), haplotype diversity (Hd), average nucleotide differences (K), and nucleotide diversity (π), were calculated using DnaSP v. 6.12.03 (Rozas et al. 2017). Genealogical relationships among species based on COI were inferred using a TCS Network in PopART v. 1.21 (http://popart.otago.ac.nz).

*Amynthas dandongensis* sp. nov. is observed to have no male pores and prostate glands (see below), a characteristic of earthworms that reproduce parthenogenetically. Thus, to further validate the new species’ identification and explore its phylogenetic position, its mitogenome was sequenced and visualized. The mitogenomic data were obtained using the next-generation sequencing with a paired-end 150 bp strategy on the platform of DNBseq platform in Novogene (Beijing, China). Clean data were filtered from the raw data following the steps of Zhao et al. (2022). The whole mitogenomic sequence was assembled and annotated by MitoZ v. 2.4 (Meng et al. 2019) and checked manually by comparing with the published mitogenomes of other available *Amynthas* species. Mitogenomic phylogenetic analyses based on the dataset of 13 protein-coding genes (PCGs, accession numbers see Table 1) that extracted from the GenBank file using the script gbseqextractor_v2.py (Meng et al. 2019), were performed using the Bayesian inference (BI) and maximum likelihood (ML) methods with a concatenated and partitioned strategies. BI was performed using MrBayes 3.2.7a (Ronquist et al. 2012); one million generations were run to achieve the average standard deviation of split frequencies less than 0.01. ML analysis was performed in RAxML 8.0 (Stamatakis 2014) using the default rapid hill-climbing algorithm and the GTRGAMMA model to search for the best tree, and the clade support was assessed using 1,000 rapid bootstrap replicates. *Polypheretima andresi* Aspe, 2025 was set as the outgroup in both BI and ML analyses.

## ﻿Taxonomy

### ﻿Family Megascolecidae Rosa, 1891

**Genus *Amynthas* Kinberg, 1867**

#### ﻿ Amynthas dandongensis

Taxon classificationAnimaliaOligochaetaMegascolecidae

Han & Zhao sp. nov.

8618A692-397C-540B-B620-562A8E8DDC31

https://zoobank.org/959B777C-7ACB-4C94-B046-85F6B48D931A

Fig. 1

##### Type material.

***Holotype***: • 1 clitellate (533R57_01) from Jinjiangshan Park, Dandong Prefecture, Liaoning (40.1312°N, 124.3746°E, 40 m elev.), China, collected by Huifeng Zhao, Yanmeng Bi and Shixiong Ma, 2023-07-26. ***Paratypes***: • 13 clitellates (533R58_01, 02; 533R60_01, 02; 533R62_01−04; 533R63_01; 533R64_01−03) with the same information with as the holotype. All the specimens of the new species have been deposited in Langfang Normal University, Hebei, China (C-HLU).

##### Diagnosis.

Small worm with size 41–57 mm by 1.0–2.5 mm (clitellum width). Segment numbers 86–104. Spermathecal pores four pairs, 5/6–8/9, medio-ventral, nearly inconspicuous. Spermathecae four pairs in VI–IX, ampulla oval; ampulla duct thick and stout, abruptly narrower towards ectal end; diverticulum with slender and long duct and ovoid receptacle; receptacle 1/3 length of the diverticulum duct. Diverticulum is the same length or slightly longer than the main pouch. Intestinal caeca simple, originating in XXVII extending anteriorly to XXI. Male pores and genital markings absent.

##### Description.

**Figure 1.**
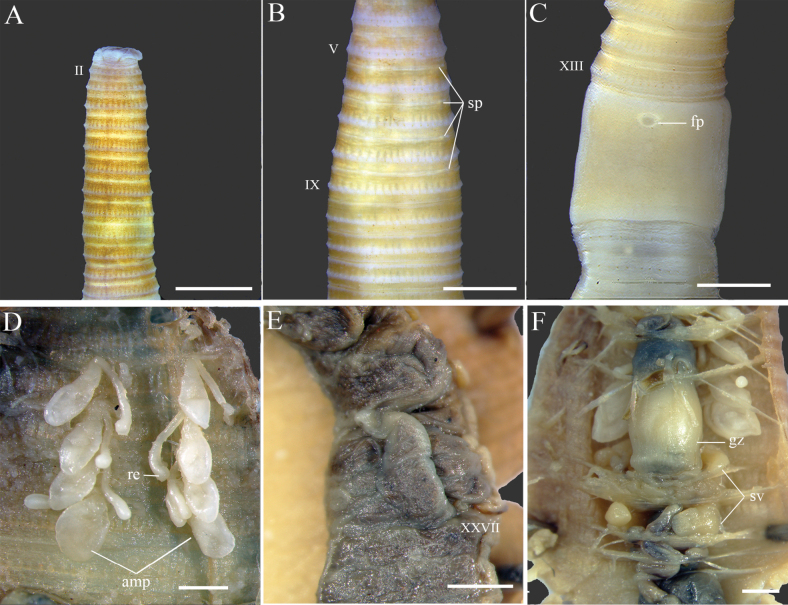
*Amynthas dandongensis* sp. nov. (specimen ID: 533R57_01, PV453020). **A.** Dorsal view of prostomium; **B.** Spermathecal pores; **C.** Female pore; **D.** Spermathecae; **E.** Left intestinal caecum; **F.** Seminal vesicles and gizzard. Abbreviations: amp = ampulla, fp = female pore, gz = gizzard, re = receptacle, sp = spermathecal pore, sv = seminal vesicle. Scale bars: 1 mm.

***External characters*.** Length 41–57 mm, clitellum width 1.0–2.5 mm, narrower toward pre-clitellar region (*n* = 5). Color of preserved specimens may vary from shades because of the duration of preservation but generally dorsum region is brown to yellowish brown, while ventrum part is pale yellow. Segment numbers 86–104. Prostomium epilobous (Fig. 1A). First dorsal pore on 12/13. Clitellum pale brown, annular and raised at XIV–XVI; setae or dorsal pores and intersegmental furrow absent. Setal arrangement perichaetine; setae number 32–35 (V), 34–37 (VII), 40–45 (XIII), 48–52 (XX). Spermathecal pores four pairs, 5/6–8/9, medio-ventral, nearly inconspicuous with 0.20–0.22 C apart ventrally. Male pores, pre-clitellar and post-clitellar genital markings absent (Fig. 1B). Female pore single, circular in a slightly concave porophore medioventral at XIV (Fig. 1C).

***Internal characters*.** Septa 5/6/7 slightly thickened, 7/8 thin, 8/9/10 absent, 10/11/12/13/14 thin. Gizzard within VIII–X, large and barrel-shaped (Fig. 1F). Intestine enlarged from XV. Intestinal caeca are simple, originating in XXVII and extending anteriorly to XXI (Fig. 1E). Esophageal hearts three pairs in X–XIII.

Spermathecae four pairs in VI–IX. Ampulla oval; ampulla duct thick and stout, abruptly narrower towards ectal end (1.3–1.8 mm long); diverticulum with slender and long duct and ovoid receptacle (1.5–2.0 mm long); receptacle 1/3 length of the diverticulum duct. Diverticulum is the same length or slightly longer than the ampulla plus duct (Fig. 1D).

Male sexual system holandric, testes sac well-developed, two pairs in X and XI. Seminal vesicles, two pairs in XI and XII. Prostate glands and accessory glands absent.

##### Distribution in China.

Liaoning Province, Northeast China.

##### Habitat.

Forest/Urban Park.

##### Etymology.

The specific name refers to the type locality, Dandong Prefecture; adjective.

##### Remarks.

*Amynthas dandongensis* sp. nov. belongs to the *A. corticis* species group (previously named the “*diffringens*” species group), characterized by having four spermathecal pores in 5/6/7/8/9. There was a total of 99 recorded species in the species group before 1972 (Sims and Easton 1972). Several species have been categorized as junior synonyms of *A. corticis* Kinberg, 1867, such as *A. diffringens* (Baird, 1869), *A. divergens divergens* (Michaelsen, 1892), *A. yunnanensis* (Stephenson, 1912) and *A. heterochaetus* (Michaelsen, 1891) by Blakemore (2004). Subsequently, an addition of 47 species/subspecies were reported: 12 species/subspecies were described from mainland China (Chen et al. 1975; Chen and Xu 1977; Sun et al. 2013; Sun et al. 2018; Dong et al. 2019; Li et al. 2024; Jin et al. 2024), eight species from Hainan Island China (Sun et al. 2012, 2013), 17 species from Taiwan Island China (Tsai et al. 2001; James et al. 2005; Chang et al. 2007; Tsai et al. 2007, 2010; Wang and Shih 2010; Shen 2012; Shen et al. 2013, 2014, 2019), eight species from South Korea (Hong and James 2001; Hong and Kim 2002; Blakemore 2012; Blakemore and Lee 2013), and three species from Laos (Hong et al. 2024).

Table 2 presents a comparison of some members of the *A. corticis* species group in China with similar or overlapping sizes. The new species, aside from its lack of male pores, prostate glands, and genital markings, is unique from the rest of the members of the group by having a smaller body size (41–57 by 1.0–2.5); the first dorsal pore on 12/13 (vs 10/11 in *A. meishanensis* Chang, Lin, Chen, Chung & Chen, 2007; 5/6 in *A. nanshanensis* Shen, Tsai & Tsai, 2003, *A. taoyuanensis* Qiu & Jin, 2024 and *A. libratus* Tsai & Shen, 2010; 5/6 or 6/7 in *A. pavimentus* Tsai & Shen, 2010 and *A. penpuensis* Shen, Tsai & Tsai, 2003; pore-like marking on 7/8 in *A. manicatus decorosus* (Gates, 1932); 13/14 in *A. tortuosus* Qiu & Dong, 2019; rectangular gizzard in VIII–X (vs VII–X in *A. meishanensis*, *A. pavimentus* and *A. libratus*; IX–X in *A. nanshanensis* and *A. penpuensis* and *A. taoyuanensis*); and having simple intestinal caeca in XXVII–XXI (vs simple in XXVII–XXIV in *A. meishanensis*, *A. taoyuanensis* and *A. tortuosus*; XXVII–XXV in *A. nanshanensis*; XXVII–XXIII (XXIV) in *A. pavimentus* and *A. libratus*; XXVII–XXV (or XXIV) in *A. penpuensis*; compound, XXVII–XXII or XXIII in *A. manicatus decorosus*. *Amynthas recavus* Yuan & Zhang, 2019 also possesses dorsal pore on 12/13 yet with a larger size than the new species, and also possesses male pores and genital markings. Generally, the obvious distinction of the new species among other members of the *A. corticis* species group, regardless of the overlapping body sizes and dorsal pore on 12/13 is its lack of male pores and genital markings, which are typical among the other members of the group.

**Table 2.** Comparison of species belonging to the *A. corticis* species group in China. The species are listed in order of size from smallest to largest.

Characters	*A. meishanensis*	*A. manicatus decorosus*	*A. dinghuensis*	*A. dandongensis* sp. nov.	*A. nanshanensis*	*A. taoyuanensis*	*A. pavimentus*	*A. libratus*	*A. tortuosus*	*A. penpuensis*	*A. recavus*
Length	38–65	40	40–52	41–57	41–89	41–120	51–121	55–72	55–86	55–104	58–64
Width	2.7–3.5	2.5		1.0–2.5	2.2–3.0	3.5–4.5	1.98–3.96	2.31–3.03	2.5–2.8	2.2–3.2	2.1–2.3
No. of segments	51–113	60	75–87	86–104	50–104	61–103	61–103	62–71	-	62–104	82–84
1^st^ dorsal pore	10/11	pore-like marking from 7/8	11/12 or 12/13	12/13	5/6	10/11	5/6 or 6/7	5/6	13/14	5/6 or 6/7	12/13
Male pores	present	present	present	absent	present	present	present	present	present	present	present
Genital markings	present	present	present	absent	present or absent	present	present	present	present	present	present
Spermathecae ampulla	peach-shaped or oval	ampulla same length as duct	elongated oval-shaped (varied)	oval-shaped	peach-shaped	oval-shaped	round to oval-shaped	round or oval-shaped	ampulla slender heart-shaped	peach-shaped	elongated oval-shaped
Diverticulum	straight stalk as long as the receptacle with peach-shaped receptacle	may be looped into regular zigzag or not looped	slender stalk and oval-shaped receptacle	slender and long duct, with ovoid receptacle	slender stalk and oval receptacle	stalk 2/3 of main pouch, bag-shaped receptacle	slender stalk and small oval-shaped receptacle	slender stalk and small oval-shaped receptacle	swollen, S-shaped twisted receptacle	slender stalk and small oval receptacle	~ 3/5 of main pouch, ovoid-shaped receptacle
Prostate gland	paired in XVIII, large w/ thick duct	paired in XVIII, extending XVII–XIX	paired in XVIII, smooth and lobed extending XIV–XX	absent	paired in XVIII, extending XVII–XX	degenerated, duct U-shaped inserting in XVIII	paired in XVIII, large, rectangular-shaped, extending XVI (XVII)–XIX (XX)	paired in XVIII, large, lobed, extending XVII–XX	paired in XVIII, extending XVII–XXII, coarsely lobate	paired in XVIII, extending XVII–XX	1/2XVII–XIX, relatively small, one lump
Gizzard	VII–X	?	large in VIII–X	VIII–X	round, IX–X	IX–X	large, round in VII–X	large, rectangular, VII–X	ball-shaped, VIII–X	round, IX–X	VIII–X, ball-like
Intestinal caeca	simple, XXVII–XXIV	compound, XXVII–XXII or XXIII	simple with a round, white end, XXVII– XXII	simple, XXVII–XXI	simple, XXVII–XXV	simple, XXVII–XXIV	simple, XXVII–XXIII (XXIV)	simple, XXVII–XXIII (XXIV)	simple, XXVII–XXIV	simple, XXVII–XXV (XXIV)	simple, XXVII, end in XXV

The new species is morphologically most similar to *A. sangumburi* (Hong & Kim, 2002) from Jeju Island, South Korea (Table 3) in terms of size range (41–57 mm by 1.0–2.5 mm in *A. dandongensis* sp. nov. and 46–68 mm by 2.3–2.5 mm in *A. sangumburi*); and having an ovate-shaped ampulla, intestinal origin in XV, and lacking genital markings. However, all the collected specimens of the new species lack male pores and prostate glands (vs present, centered on 0.8 mm circular flat porophores extending to lateral margins of ventrum in XVIII with prostate glands large within XVII–XX in *A. sangumburi*); and have more segments (86–104 vs 58–79). In addition, a previously recorded species from northeastern China, *A. carnosus* (Goto & Hatai, 1899), with four pairs of spermathecae in 5/6–8/9, differs from the new species in the body size, spermathecae shape, genital markings, and the male pore region. Currently, no other known species is morphologically similar to *A. dandongensis* sp. nov.

**Table 3.** Summary comparison between *Amynthas dandongensis* sp. nov. from China and *A. sangumburi* from Korea.

Descriptions	*A. dandongensis* sp. nov.	*A. sangumburi*
No. of specimens	14	5
Length	41–57	46–68
Width	1.0–2.5	2.3–2.5
Color	dorsum brown to yellowish brown, while ventrum part is pale yellow	dorsum pale brown
Segment No.	86–104	58–79
1^st^ dorsal pore	12/13	12/13
Setae	32–35 (V), 40–45 (XIII), 48–52 (XX)	39 (VII), 43 (XX); 3–4 between male pores
Spermathecal pores	5/6–8/9, medio-lateral, nearly inconspicuous with 0.20–0.22 C apart ventrally	5/6–8/9, lateral, very small
Male pores	absent	centered on 0.8 mm circular flat porophores extending to lateral margins of ventrum in XVIII
Genital markings	absent	absent
Septa	Septa 5/6/7 slightly thickened, 7/8 thin, 8/9/10 absent, 10/11/12/13/14 thin	5/6, 6/7 thick, 7/8 some muscle, 8/9 absent; 9/10 if present is part of testis sac X, 10/11–13/14 thin
Spermathecae	four pairs in VI–IX ampulla oval; ampulla duct thick and stout, abruptly narrower towards ectal end (1.3–1.8 mm long); diverticulum with slender and long duct and ovoid receptacle (1.5–2.0 mm long); receptacle 1/3 length as with the diverticulum duct	four pairs of spermathecae in VI–IX; ampulla cylindrical (VI, VII) to sagittate or ovate; ducts short, not muscular, diverticuum stalked, gradually widening distally
Gizzard	VIII–X	VIII–X
Seminal vesicles	two pairs in XI and XII	two pairs in XI, XII
Testis	paired in X, XI	paired in X, XI
Intestinal origin	XV	XV
Prostate glands	absent	in XVIII, large within XVII–XX; thick ducts of median length, nearly straight, both glandular portions consist of 2 or 3 main lobes, each lobe divided into leaflets
Intestinal caeca	simple, in XXVII and extending anterior to XXI	simple, in XXVI/XXVII, and extending anterior to ~ XXIII/XXIV
Accessory glands	absent	absent

### ﻿Genus *Metaphire* Sims & Easton, 1972

#### ﻿ Metaphire agrestis

Taxon classificationAnimaliaOligochaetaMegascolecidae

(Goto & Hatai, 1899)

AFE0A675-E8A3-5B44-84DA-04869B5E44C6

Figs 2, 3

Perichaeta agrestis Goto & Hatai, 1899: 17, 24.
Pheretima agrestis — Michaelsen 1900: 313; Yamaguchi 1930: 51; 1962: 25; Kobayashi 1938: 141; Hatai 1930: 651; Howell 1939: 231. Gates 1953: 5; 1954: 224; 1958: 1, 31; 1963: 11; 1982: 38.
Amynthas agrestis —Beddard 1900: 637; Sims and Easton 1972: 235. Reynolds 1978: 119, 127; 2010: 143; 2011: 269; 2014: 116; 2022:110. Easton 1981: 51. Reynolds and Wetzel 2004: 88; 2008: 179. Hong and Kim 2005: 130; 2009: 137. Blakemore 2010: 429; 2013a: 56, 57; Chang et al. 2016: 503. Minamiya 2017: 14; Shekhovtsov et al. 2018: 11. Schall et al. 2023: 58.
Metaphire agrestis —Blakemore 2003: 7, 28 (syn. *striata*). Uchida and Ihara 2003: 34. Uchida and Kaneko 2004: 37. Iki and Ohba 2022: 42. Sato et al. 2023: 5, 6, 8.
Pheretima hataii —Ohfuchi, 1937: 13 ; Ohfuchi, 1938: 178.
Metaphire hataii —Sims and Easton 1972: 238. Easton, 1981: 58. Blakemore, 2007a: 46; 2008: 113; 2010a: 19; 2012: 19.

##### Material examined.

• 5 clitellates (533R71_02, 533R72_03, 533R74_01, 533R74_02, and 533R75_02) from Jinjiangshan Park, Dandong Prefecture, Liaoning (40.1312°N, 124.3746°E, 40 m elev.), collected by Huifeng Zhao, Shixiong Ma, Yanmeng Bi, and Yufeng Zhang, 2023-07-26. All other specimens have been deposited in C-HLU.

##### Diagnosis (modified from Reynolds 2022).

Size 70–200 mm length by 5–8 mm (clitellum width). Segment numbers 63–110. Spermathecal pores three pairs in 5/6/7/8. Pre-clitellar genital markings present or absent; when present, ventral, areas of slight epidermal modification on VII and/or VIII, occasionally on VI and IX, unpaired and median or symmetrically paired, forming setal gaps, epidermis finely wrinkled or crosshatched, sometimes darker in color in live specimens. Male pores usually absent; when present, small, transversely slit-like. Post-clitellar genital markings usually absent; when present, single, large circular pad, pre-setal on XVIII, just median to male pores, with a concave center surrounded by a narrow but distinct, raised rim, reaching posteriorly slightly behind the setal line on XVIII and anteriorly to the setal line on XVII. Spermathecae present or absent; when fully present, three pairs in VI–VIII, duct shorter than ampulla; diverticulum longer than the main pouch. Prostate glands present or absent; when present, extending through some or all of XVI–XXIII, ducts in XVIII. Accessory gland mostly absent, when present, large and paired in front of the setal line on segment XVIII. Intestinal caeca paired in XXVII extending anteriorly to XX, digitate.

##### Description of Chinese specimens in this study.

**Figure 2.**
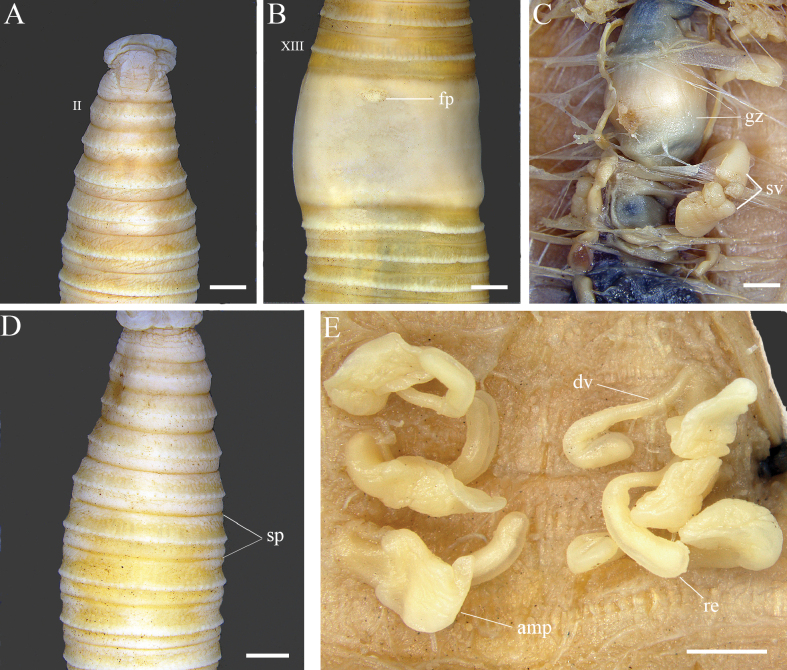
*Metaphire agrestis* (specimen ID 533R71_02, PV453015). **A.** Dorsal view of prostomium; **B.** Female pore; **C.** Seminal vesicles and gizzard; **D.** Spermathecal pores; **E.** Spermathecae. Abbreviations: amp = ampulla, fp = female pore, gz = gizzard, re = receptacle, sp = spermathecal pore, sv = seminal vesicle. Scale bars: 1 mm.

***External characters*.** Length 80–94 mm by width 5.0–8.0 mm (*n* = 5). Color of live specimens reddish-brown. Color of preserved specimens may vary in shades because of the duration of preservation but generally brownish-yellow (with blackish highlights) throughout with milk gray clitellum. Number of segments 100–108. Prostomium epilobous (Fig. 2A). First dorsal pore on 12/13. Clitellum annular at XIV–XVI; setae or dorsal pores and intersegmental furrow absent. Setal arrangement perichaetine; setae number 36–40 (V), 52–65 (VII), and 55–72 (VIII). Female pore single in medio-ventral at XIV (Fig. 2B). Spermathecal pores three pairs, 5/6–7/8, ventro-lateral, 0.28–0.30 C apart ventrally. Pre-clitellar genital markings absent (Fig. 2D). Male pores and post-clitellar genital markings absent.

**Figure 3.**
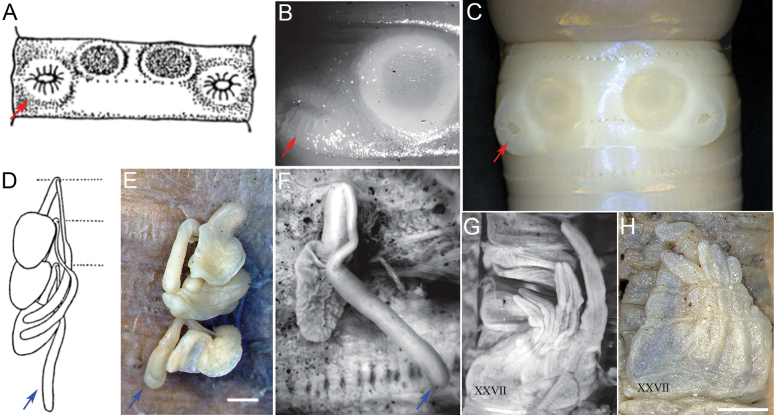
*Metaphire agrestis* specimen comparison. **A, B.** Male pore (red arrows) with post-clitellar genital markings; **D, E, F.** Spermathecae and diverticulum (blue arrows); **C, G.** Right and left intestinal caeca. (**A, D**) sourced from the original drawings of *Pheretima hataii* (= *M. agrestis*) from Japan (Ohfuchi 1937); (**B, F, G**) from the USA (Chang et al. 2016); (**C, E**) from China in this study. Scale bars: 1 mm.

***Internal characters*.** Septa 4/5/6 quite thickened, 6/7/8 thin and fibrous, 8/9/10 absent, 10/11/12/13 thin. Gizzard within VIII–IX (Fig. 2C). Intestine enlarged from XIV. Intestinal caeca manicate with five finger-like sacs, originating in XXVII and extending anteriorly to XX (Fig. 3H). Last pair of hearts in XIII.

Spermathecae present, three pairs in VI–VIII ampulla ovate, surface flattened and wrinkled, 1.5–1.8 mm long; ampulla duct long, stout, 1.1–1.3 mm long, with a swollen basal portion; diverticulum originating from below the swollen portion of the spermathecal duct, stalk slender with a swollen long receptacle; diverticulum longer than the main pouch (Fig. 2E). Seminal vesicles two pairs in XI and XII, well developed. Prostate glands and accessory glands absent.

##### Global distribution.

Cosmopolitan/Exotic. USA (Gates 1953, 1954, 1958, 1963, 1966, 1982; Davies 1954; Reynolds 1978, 2010, 2011, 2015a, 2015b; Callaham et al. 2002; Görres and Melnichuk 2012; Chang et al. 2016; Görres et al. 2017; Chang et al. 2021), Canada (Reynolds 2014, 2023), Japan (Kato 1972; Minamiya 2014), South Korea (Kobayashi 1935; Song and Paik 1969, 1970a, 1970b, 1971, 1973; Hong and Kim 2005; Hong and Kim 2009; Blakemore 2013a, 2013b), North Korea (Kobayashi 1935, 1938); Russia (Shekhovtsov et al. 2018), and China (this study).

##### Distribution in China.

Liaoning Province, northeast China.

##### Habitat.

Urban parks.

##### Biology.

Known as “jumping worms”, “snake worms”, or “crazy worms” because of their erratic thrashing behavior when disturbed.

##### Remarks.

Relevant literature revealed that *M. agrestis* (= *A. agrestis*) has not been previously recorded in China (Blakemore 2007b, 2009; Xu and Xiao 2011). *Metaphire agrestis* is widely distributed in East Asia such as Japan (Kato 1972; Minamiya 2014), Korean Peninsula (Kobayashi 1935, 1938; Hong and Kim 2005, 2009; Blakemore 2013a, b) and in the northern islands (Kunashiri Island and Yuri Island) of Russia (Shekhovtsov et al. 2018). It is known to be mostly dominant in Japan and Korean peninsula, where it is considered as native. *Metaphire agrestis* is also considered as one of the invasive pheretimoid earthworms in the USA and Canada where it is rather known as *A. agrestis* (Chang et al. 2016, 2021; Keller et al. 2017; Reynolds 1978, 2023).

Additional descriptions of *M. agrestis* (= *A. agrestis*) were made by Gates (1982) and Reynolds (1978). Subsequently, *Pheretima hataii* (= *M. hataii*), which was described by Ohfuchi (1937), possesses some distinguishing features (having red pre-clitellar genital markings only on VII, invariably possessing one pair of male pores and genital markings pre-setal on XVIII and having paired genital markings pre-setal on XVIII) from that of *M. agrestis* (having red pre-clitellar genital markings on both VII and XII, has a very low rate of species with male pores). Given these insufficient descriptions for it to be recognized as a distinct species, Uehira (1978) suggested the possibility of its synonymization to *M. agrestis* (Minamiya 2014). Likewise, Minamiya (pers. comm. 18 Nov 2025) considered *P. hataii* (= *M. hataii*) as a synonym of *M. agrestis*, stating that the distinguishing characters described in the original record for *P. hataii* (= *M. hataii*) are included within the individual variation of *M. agrestis*.

A summary of comparison for *M. agrestis* is presented in Table 4. All Chinese specimens of *M. agrestis* were found to have no male pores, which pertains to parthenogenetic reproduction. Meanwhile, specimens of the USA are also usually without male pores. If present, male pores are small, transversely slit-like (Chang et al. 2016), and the distance between male pores is ~1/3 of the body circumference (Kobayashi 1938; Reynolds 1978). Reynolds (1978, 2023) described the male pores as being “minute and located on an eversible papilla, inside a transverse slit-like parietal invagination on an elevated porophore.” It is also worth noting that Reynolds (2023) has used the same photographs of *A. agrestis* reported by Chang et al. (2016). Post-clitellar markings usually absent, if present, they are paired, pre-setal on the XVIII, measuring 0.8–2.2 mm.

**Table 4.** Comparison of characters among *M. agrestis* (Goto & Hatai, 1899) from Japan, South Korea, North America, and China.

Species	*Perichaeta agrestis* (original description)	*A. agrestis*	*A. agrestis*	*A. agrestis*	*M. agrestis*
Source	Goto and Hatai 1899	Chang et al. 2016	Reynolds 2023	Blakemore 2013	this study
Locality	Japan	USA	Canada	Korea	Northeast China
Length	100–160	70–160	70–200	100–140	80–94
Segments	80–96	63–110	67–110	105	100–108
diameter	5–8	5–8	5–8		5–8
1^st^ dorsal pore	11/12 or 12/13	12/13	12/13	11/12 or 12/13	12/13
Setal number	36 (spermathecal region), 40+ (more posteriorly)				36–40 (V), 52–65 (VII), 55–72 (VIII)
Spermathecal pores	three pairs, in 5/6/7/8	three pairs, in 5/6/7/8 or variously missing	minute and superficial in 5/6–7/8, ~1/3 circumference (C) apart	three pairs, in 5/6/7/8	three pairs, in 5/6/7/8 (0.28–0.30 C apart ventrally)
Male pores	absent	usually absent; when present, small, transversely slit-like	absent; when present minute and located on an eversible papilla	absent	absent
Pre-clitellar genital markings	absent, however, there are two pairs of slightly elevated squarish patches of a pale brown color inside the spermathecal pores, one in VII and the other in VIII	absent; when present, ventral, areas of slight epidermal modification on VII and/or VIII, unpaired and median or symmetrically paired, forming setal gaps	usually lacking	dark patches on elongate on VII and paired in VIII in some specimens	absent, however a slightly pale brown patch around the midventral area of the spermathecal pores
Post-clitellar genital markings	absent	usually absent; when present, single, large circular pad, pre-setal on XVIII, median to male pores	a pair of large presetal papillae in18 (0.8–1.8 mm in diameter), median to the male porophores	absent	absent
Spermathecae	three pairs in VI–VIII	present or absent; when fully present, three pairs in VI–VIII	three pairs in VI–VIII		three pairs in VI–VIII
Ampulla and duct		duct shorter than ampulla	dorsoventrally flattened with thin wall and numerous wrinkles; duct with thick wall, shorter than ampulla		ovate, surface flattened and wrinkled, (1.5–1.8 mm); duct long, stout, (1.1–1.3 mm)
Diverticulum	diverticula longer than the main sac, but almost straight or very slightly winding	diverticulum longer than duct and ampulla combined.	longer than the main pouch, with sausage-shaped seminal chamber		longer than the main pouch; stalk slender with a swollen long receptacle
Prostate glands	absent	present or absent; extending through some or all of XVI–XXIII	usually absent, but when present in XVI–XX only when male terminalia are present	absent	absent
Seminal vesicles			in XI and XII, filling coelomic cavities of those segment	large in XI and XII	large in XI and XII
Hearts			XI–XIII		
Intestines	XV				
Gizzard	VIII–IX				VIII–IX
Intestinal caeca	manicate, paired in XXVII bearing seven secondary caeca, the dorsal most being the longest reaching to XXIV		manicate, paired in XXVII and extending anterior to XXIV, with six to nine secondary caeca, the dorsal most being the longest	manicate; paired in XXVII	
Accessory glands	absent		low and confined to or extending only slightly above the parietes		

Chang et al. (2016) provided a photograph of a specimen of *A. agrestis* with a single male pore and a genital marking (Fig. 3B). This figure matches with the original illustration of the male pore and genital markings of *P. hataii* (= *M. agrestis*) (Fig. 3A) by Ohfuchi (1937) and with a *M. agrestis* specimen from Japan (Fig. 3C; photograph provided by Minamiya, pers. comm. 18 Nov 2025) although the latter two figures have paired male pores and genital markings. Meanwhile, the spermathecae of the Chinese specimens (Fig. 3E) match their corresponding specimen counterparts from Japan (Fig. 3D) and the USA (Fig. 3F); whereas the intestinal caeca (Fig. 3H) match those from the USA (Fig. 3G).

### ﻿The generic re-assignment of the species *agrestis* to *Metaphire*

Sims and Easton (1972) stated that the only distinguishing character of *Amynthas* and *Metaphire* is the absence of copulatory pouch in the former, while the presence of copulatory pouch in the latter. However, the homology among copulatory pouch has not been clear since various definitions of the character have been proposed. Some authors prefer to restrict *Metaphire* to species with well-developed copulatory pouch (protruding into the coelom) (James 2005; James et al. 2005), whereas others also consider intramural chambers and even shallow indentations to be copulatory pouches (Tsai et al. 2009; Shen et al. 2019). Chang et al. (2014) and Shen et al. (2019) have given further discussions and views on this topic. While Chang et al. (2014) doubted that species with an intramural form of copulatory pouch could be assigned to either *Amynthas* or *Metaphire*; Shen et al. (2019) argued that regardless of whether a “copulatory pouch” is intramural, intra-coelomic or well-characterized, the species possessing this structure should not be placed under *Amynthas* (as species in this genus are readily recognized by the absence of a copulatory pouch or by having superficial male pores).

In the case of *Pheretima* and *Metaphire*, Sims and Easton (1972) defined the type of invagination to be the same in the two genera. James et al. (2005) supported the restriction of *Metaphire* “to those species distinguishable from *Pheretima* only by the absence of nephridia from the spermathecal ducts (Sims and Easton 1972)”. This required the presence of a well-developed copulatory pouches protruding into the coelom (as in *Pheretima*) (James et al. 2005). On the other hand, Aspe and James (2014), in their report of new *Pheretima* species from Mindanao, Philippines, proposed that the “relative size of the copulatory bursae” may be a distinguishing character between *Pheretima* and *Metaphire*. Consequently, those species (reported in their study) which tend to have more prominent dome-shaped, intra-coelomic copulatory bursae than those in *Metaphire* were placed in genus *Pheretima* (e.g., Ohfuchi 1938, 1957; Tsai et al. 2004; Bantaowong et al. 2011). This also implies that those species with “less prominent” intra-coelomic copulatory bursae (without nephridia from the spermathecal ducts) should be placed in *Metaphire*. Furthermore, with regards to species identification, irrespective of the definition of the copulatory pouch and the assignment of a new species to one of the two genera, comparison of species should always be made with the most similar species in both *Amynthas* and *Metaphire* to avoid possible synonymy (Shen et al. 2019).

As with the generic assignment of *agrestis* to *Amynthas*, we find those previous taxonomic accounts (Chang et al. 2016; Reynolds 1978, 2023) questionable, because the descriptions of the male pores of their *A. agrestis* specimens are more likely to fall into the category defined as a copulatory pouch (as in the genus *Metaphire*). Terms such as “transverse slit-like” (Chang et al. 2016), “eversible papilla”, “transverse slit-like parietal invagination on an elevated porophore” (Reynolds 1978, 2022, 2023) would actually imply openings of copulatory pouches (=secondary male pores) (Shen et al. 2019). Gates (1982), in his description of the male pores of *P. agrestis*, likewise uses the terms “irregular protuberance… withdrawn into the body wall”, “secondary aperture with finely wrinkled margin” and “transverse slit-like”, which obviously imply the presence of a copulatory pouch. He then stated that the primary male pores were almost certainly not superficial and that *agrestis* does not belong in any genus defined as having superficial male pores. Hence, it could not be placed under genus *Amynthas*.

Furthermore, the classification of *agrestis* to genus *Amynthas* by Blakemore (2007a, 2010, 2012) was through his personal observation of a “newly inspected material” collected from Ibaraki Prefecture in Japan wherein the male pores were described to be “small and superficial in setal arc below tumid genital markings” (fig. 3 in Blakemore 2010). However, Blakemore (2014) later questioned these findings and noted that because of the difference in intestinal caeca, the specimen that is believed to be *A. agrestis* may belong to a different species. In addition, the placement of *agrestis* to *Amynthas* by Ito et al. (2011) was invalidated as the genital markings were mistaken as male pores by Ishizuka (2001), and the specimen does not have a male pore (Minamiya 2014).

Given the above arguments and information coupled with our examination and comparison of the three illustrations of the male pore region of *A. agrestis* as reported by Chang et al. (2016) and with that of the *M. agrestis* (= *M. hataii*) illustration from Ohfuchi (1937) and a photograph by Minamiya (pers. comm.), which visibly bear the presence of an invagination (= copulatory pouch) in the male pore area (not superficial), we stand that the *agrestis* species should be placed in the genus *Metaphire*.

Regardless of differing opinions on the definition of the *Metaphire* and *Amynthas*, several molecular studies have revealed that both genera are non-monophyletic (e.g., James 2005; Chang et al. 2008; Zhao et al. 2015; Zhang et al. 2015; Aspe and James 2018; Aspe et al. 2025). However, Chang and James (2011) suggested the use of longer sequences from both mitochondrial and nuclear genes (> 2,000 bp) to obtain a more accurate phylogenetic tree. This can be coupled with a deeper look and re-examination of other morphological characters, which may help better discriminate the two genera. Aspe et al. (2025) suggested re-examining the male pores and the copulatory bursae of the *Amynthas* and *Metaphire* specimens by using histological analysis with support from molecular data as done in Nguyen et al. (2020, 2022) on Vietnamese pheretimoids to validate the taxonomy of these genera.

### ﻿Genetic analysis

**Figure 4.**
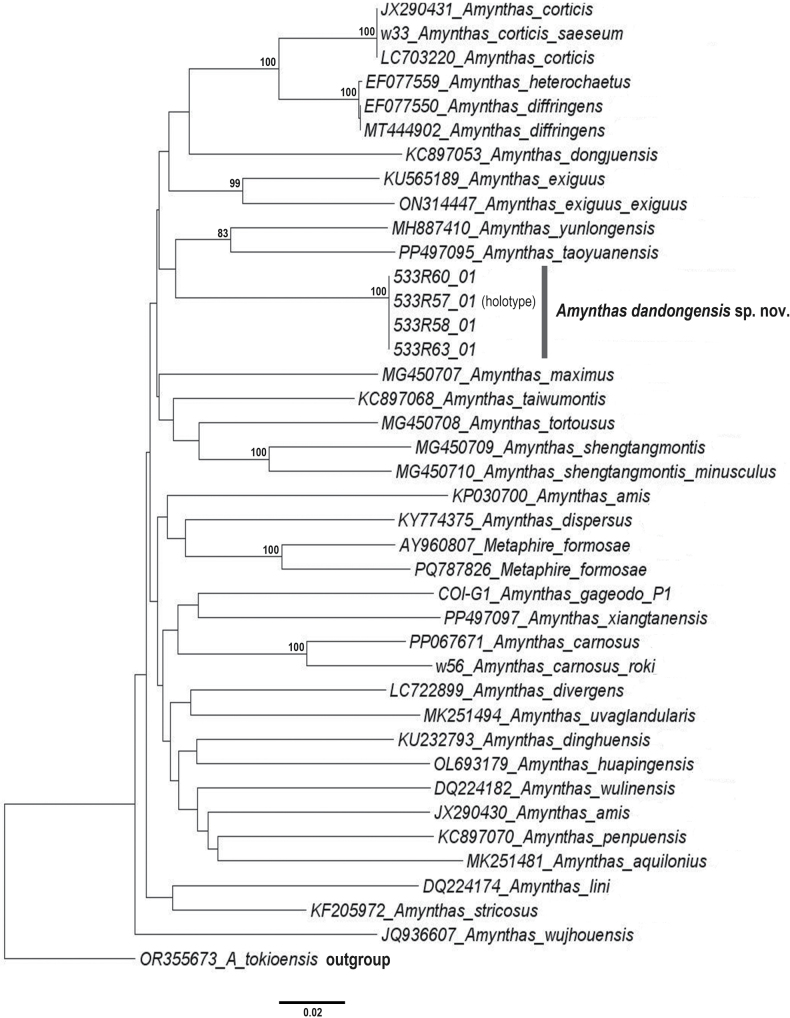
Neighbor-joining tree of *Amynthas dandongensis* sp. nov. and members of the *A. corticis* species group. The tree is based on COI gene and Kimura’s two-parameter model, and rooted using *Amynthas tokioensis*. Specimens from this study and sequences retrieved from GenBank are shown by their voucher numbers and GenBank accession numbers, respectively. Type materials are indicated in parentheses. Numbers around nodes are bootstrap values > 50.

K2P analysis of COI shows an interspecific genetic distance range of 15–22% between *A. dandongensis* sp. nov. and other species members of the *A. corticis* group (Table 5). This interspecific distance value is similar to the interspecific distance range of 14.2–23.3% (Nguyen et al. 2020), 14.7–25% (Dong et al. 2019), 16–22% (Han et al. 2024), and 15–16% (Chang and Chen 2008) reported among species within the genera *Amynthas* and *Metaphire*. Also, the genetic distance among the species members belonging to the *A. corticis* group ranges from 15% to 29%. A neighbor-joining tree based on representative COI sequences of the new species along with members of the *A. corticis* species group is also presented (Fig. 4). The COI tree has resulted in many polytomies with many species having low support values (below 50). This is expected, as resolving phylogenetic relationships among species taxa based solely on mitochondrial DNA is limited and simply not sufficient (Cameron et al. 2004; Shekhovtsov et al. 2024). Hence, the use of mitogenomic data (e.g., Sato et al. 2023; Aspe et al. 2025) has been on the rise in effectively obtaining phylogenetic trees with higher resolution. It may also be applied to species classification among pheretimoids, in which the internal structure and external pores (male pores) and spermathecal organs, which are vital characteristics for taxonomic identification, are often lost through parthenogenesis (Blakemore 2010), making species delimitation even more challenging.

**Table 5.** Percentage K2P distances of the COI gene of *M. agrestis*, *A. dandongensis* sp. nov. along with members of the *A. corticis* species group. Sequences were obtained from GenBank. (Asterisk indicates other species members of *A. corticis* species group listed in Table 1).

	Species	1	2	3	4	5	6	7
**1**	*M. agrestis*	0–1						
**2**	*A. tokioensis*	13–14						
**3**	*A. corticis*	18–19	21	0				
**4**	*A. diffringens*	18–19	20	8	0			
**5**	*A. heterochaetus*	18	20	8	0	0		
**6**	*A. dandongensis* sp. nov.	18–19	21	19	17	17	0	
**7**	other species members*	19–27	21–29	16–27	15–26	15–26	15–25	

**Figure 5.**
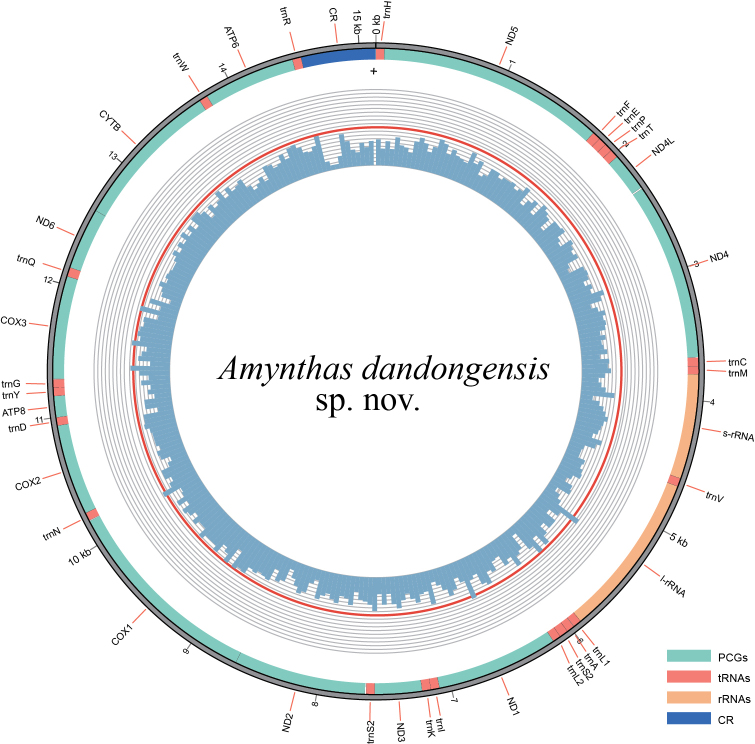
Complete mitochondrial genome of *Amynthas dandongensis* sp. nov. The inner circle indicates the GC content, and the outer circle shows the arrangement of the genes.

**Figure 6.**
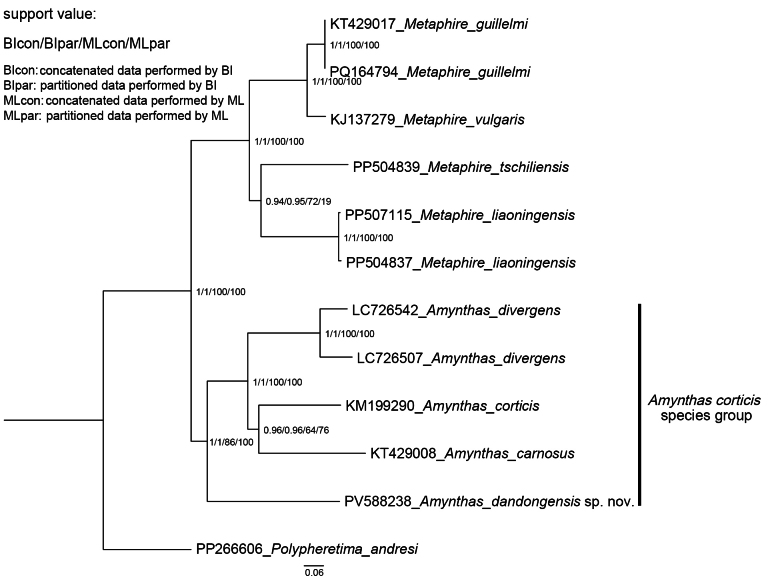
Phylogenetic tree constructed using Bayesian inference and maximum likelihood of *Amynthas dandongensis* sp. nov. with other members of the *A. corticis* species group and other pheretimoid species using mitogenomic 13 protein coding genes.

The complete and circular mitogenome of *A. dandongensis* sp. nov. is depicted in Fig. 5, and its annotation information has been submitted to GenBank (accession number: PV588238), with a gene arrangement conserved among congeners (Sato et al. 2023; Zhang et al. 2015, 2016; Zhao et al. 2025). The mitogenomic PCG-based phylogenetic tree recovered *A. dandongensis* sp. nov. as a relatively basal lineage within the *A. corticis* species group, distinct from other members with high nodal support (Fig. 6). These findings corroborate its taxonomic placement within this group and clarify its phylogenetic relationships with congeners. However, given the limited mitogenomic data currently available, the phylogenetic position of the new species still warrants further validation.

**Figure 7.**
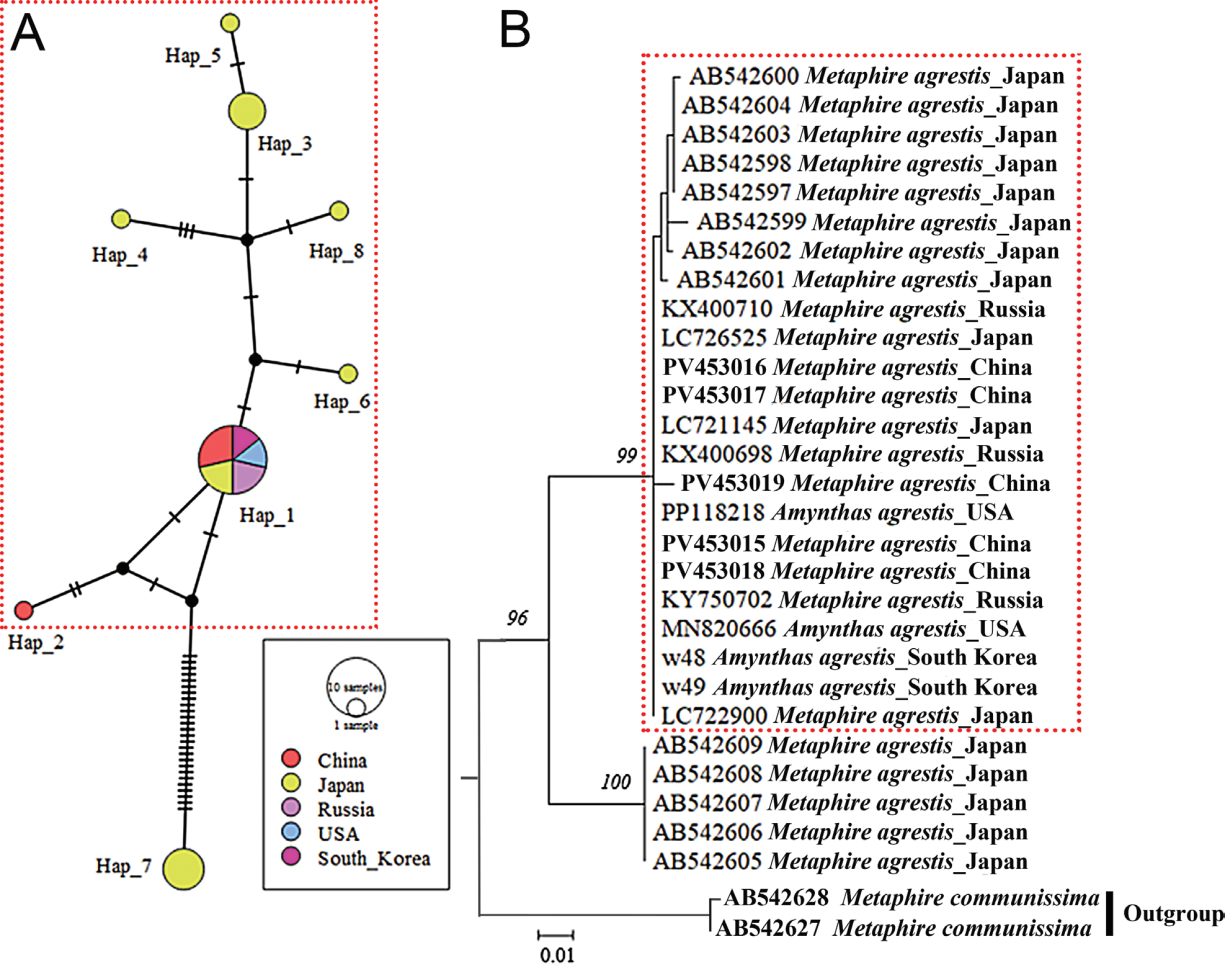
**A.** Haplotype network for *Metaphire agrestis*. Circle sizes are proportional to the number of specimens having this haplotype. Different colors in the circles indicate the distribution in different populations, the oblique lines indicate mutations between haplotypes and small black empty nodes could represent intermediate unsampled haplotypes (inferred median needed to connect the present observed haplotypes). COI sequences of Japan, Russia and the USA were retrieved from the GenBank, while those from South Korea are from Blakemore (2013a); **B.**COI tree using Maximum Likelihood method with *M. communissima* as the outgroup. Numbers near branches indicate the maximum likelihood bootstrap support. The clade comprising *M. agrestis* and ‘*A. agrestis*’ sequences and their corresponding haplotypes are enclosed in dotted red box.

Of 14 COI sequences identified as *M. agrestis* (11) and *A. agrestis* (3), three sequences of *M. agrestis* (LC722900, LC726525, and LC7229008) from Japan, three sequences of *M. agrestis* (KX400698, KY750702, and KX400710) from Russia, and one sequence of *A. agrestis* from the USA (PP118218) were 99% similar to our five sequences. In addition, two COI sequences of *A. agrestis* (IDs of w48 and w49) from South Korea (Blakemore 2013b) were also 99% similar to our specimens. The ML tree for COI sequences of *M. agrestis* and *A. agrestis* shows a monophyletic group with a high bootstrap support (BS) value of 99% (Fig. 7B) with an intra-subspecific genetic distance of 0–1%. Meanwhile, COI sequences of “*M. agrestis*” (AB542605–AB542609) from Japan have a BLAST percent similarity of 93–94% from the Chinese specimens (with a 6–7% inter-lineage genetic distance), forming a sister clade with the other monophyletic group of *M. agrestis* and *A. agrestis* specimens from other countries (with BS value of 96%). However, the given DNA sequences were not reported in a published work and there is no corresponding morphological analysis to verify species delimitation; thus, the species identity is problematic.

There are eight haplotypes within five populations of *M. agrestis* and *A. agrestis* (Tables 5, 6). One haplotype (Hap 1) was found in all five populations, and the remaining seven haplotypes (Hap 2–8) were designated as “private haplotypes” (unique to that particular region) (Sjöstrand et al. 2014) (Fig. 7A).

**Table 6.** Genetic (haplotype) diversity of the COI in *M. agrestis* from China and other countries. N, number of sequences; S, number of polymorphic sites; H, number of haplotypes; Hd, haplotype diversity; K, average number of nucleotide differences; π, nucleotide diversity.

Populations	N	S	H	Hd	K	π
Japan	19	36	7	0.81971	13.09942	0.02641
China	5	3	2	0.4000	1.2000	0.00242
South Korea	2	0	1	0.000	0.000	0.000
Russia	3	0	1	0.000	0.000	0.000
USA	2	0	1	0.000	0.000	0.000
Total data estimates	31	38	8	0.67312	9.15484	0.01846

Based on the constructed ML tree (Fig. 7B), there are two main branches of the species *agrestis* (the third branch being the outgroup). The first branch consists of *M. agrestis* and ‘*A. agrestis*’ grouped together from among five populations while the second branch consists of another group of *M. agrestis* from only one population (Japan). Seven of eight haplotypes were found in the first larger branch, which was similar to the aggregation of the overall haplotype network distribution (Fig. 7A). For most haplotypes, one (Hap 1) showed to be the center of radiation distribution. The Hap 1 was likely the most primitive haplotype (ancestral), which evolved into others.

The single haplotype (Hap 1) spanning all five populations indicates that there is no significant genetic isolation or minimal genetic differences between *agrestis* populations (regardless genus assignment) from Japan, South Korea, China, Russia, and the USA. This suggests a shared ancestry and gene flow, potentially through recent interbreeding or migration (Heimburger et al. 2023). Moreover, the ML tree and the network between haplotypes revealed that there is no significant lineage differentiation between the five *M. agrestis* and ‘*A. agrestis*’ populations (as shown in dotted red box). This further confirms the single species identity of all *agrestis* specimens from the five countries (including the *M. agrestis* reported in China, with the support of morphological data). However, the second branch identified as “*M. agrestis*” showed multiple mutations (as short lines or “edges”) from those of the main large central node (Hap 1), which implies a genetic variation.

## ﻿Conclusions

Recent surveys of pheretimoid earthworms in northeastern China have revealed new species and new records, especially in Liaoning Province. With the discovery and description of the new species and *M. agrestis* supported by molecular data, the growing taxonomic record of the pheretimoid earthworm fauna of the region has been updated. In addition, an interspecific distance range between *A. dandongensis* sp. nov. and other members of the *A. corticis* species group (15–25%) and other pheretimoid species (18–21%) is reported. This study has also demonstrated the utilization of mitogenomic data to further elucidate phylogenetic relationships between species. We correct the misplacement of *Amynthas agrestis* and clarify its genus assignment to the genus *Metaphire*, based on previous descriptions and illustrations of its previous specimens as well as new molecular data.

## Supplementary Material

XML Treatment for Amynthas dandongensis

XML Treatment for Metaphire agrestis

## ﻿Additional information

### Use of AI

No use of AI was reported.

### Author ORCIDs

Anne Charis N. Han https://orcid.org/0009-0000-7312-4985

Nannan Zhao https://orcid.org/0009-0005-4694-3121

Nonillon M. Aspe https://orcid.org/0000-0003-0025-6017

Christian Jay R. Nob https://orcid.org/0000-0003-2463-6698

Yufeng Zhang https://orcid.org/0000-0002-4066-6355

Donghui Wu https://orcid.org/0000-0001-8778-0107

Huifeng Zhao https://orcid.org/0000-0003-4243-9671

### Data availability

All of the data that support the findings of this study are available in the main text.
